# First fossil frog from Antarctica: implications for Eocene high latitude climate conditions and Gondwanan cosmopolitanism of Australobatrachia

**DOI:** 10.1038/s41598-020-61973-5

**Published:** 2020-04-23

**Authors:** Thomas Mörs, Marcelo Reguero, Davit Vasilyan

**Affiliations:** 1 Department of Palaeobiology, Swedish Museum of Natural History, P.O. Box 50007, SE-104 05 Stockholm, Sweden; 2 Bolin Centre for Climate Research, Stockholm University, Stockholm, Sweden; 3 Instituto Antártico Argentino, Campus Miguelete, 25 de Mayo 1151, 3° piso B1650HMK, San Martín, Buenos Aires, Argentina; 4 JURASSICA Museum, Route de Fontenais 21, 2900 Porrentruy, Switzerland; 5 Department of Geosciences, University of Fribourg, Chemin du musée 6, 1700 Fribourg, Switzerland

**Keywords:** Palaeontology, Biogeography, Palaeoclimate

## Abstract

Cenozoic ectothermic continental tetrapods (amphibians and reptiles) have not been documented previously from Antarctica, in contrast to all other continents. Here we report a fossil ilium and an ornamented skull bone that can be attributed to the Recent, South American, anuran family Calyptocephalellidae or helmeted frogs, representing the first modern amphibian found in Antarctica. The two bone fragments were recovered in Eocene, approximately 40 million years old, sediments on Seymour Island, Antarctic Peninsula. The record of hyperossified calyptocephalellid frogs outside South America supports Gondwanan cosmopolitanism of the anuran clade Australobatrachia. Our results demonstrate that Eocene freshwater ecosystems in Antarctica provided habitats favourable for ectothermic vertebrates (with mean annual precipitation ≥900 mm, coldest month mean temperature ≥3.75 °C, and warmest month mean temperature ≥13.79 °C), at a time when there were at least ephemeral ice sheets existing on the highlands within the interior of the continent.

## Introduction

**Figure 1:**
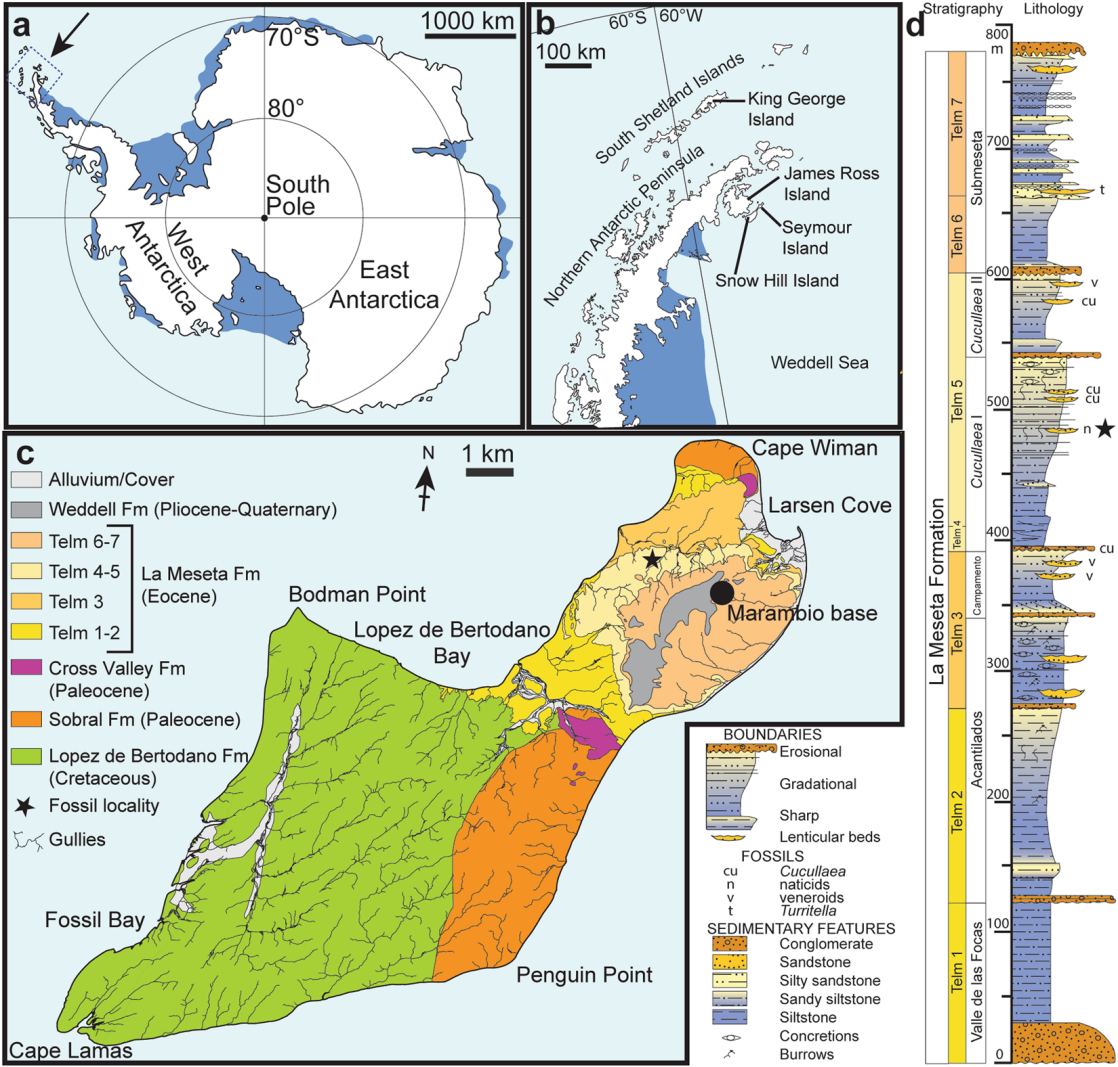
Maps and stratigraphic column of the studied area and succession. **(a)** Map of Antarctica showing the location of the study area. **(b)** Map of the northern Antarctic Peninsula showing the location of Seymour Island. **(c)** Geological sketch map of Seymour Island, showing the position of the fossil locality (asterisk). **(d)** Stratigraphic column of the La Meseta Formation on Seymour Island (from^11^) showing the position of the fossil locality (asterisk). Redrawn from^13^.

Consistent with the geological evidence, it has been hypothesized that the formation of Antarctic ice sheets predates the final break-up of Gondwana, the opening of the Drake Passage and the thermal isolation of the continent^1–3^. This is reflected by a low diversity of terrestrial mammals on the Antarctic Peninsula during the middle to late Eocene with only two species of large mammals and ten species of small mammals^4–6^ which sharply contrasts with the highly diverse marine fish fauna indicating temperate conditions in the Weddell Sea^7,8^. However, no Cenozoic ectothermic continental vertebrates (freshwater fishes, amphibians and reptiles) have been known from Antarctica so far. Here we report the discovery of a fossil ilium from Seymour Island, Antarctic Peninsula (Fig. 1a,b) which can be assigned to the lissamphibian order Anura, and a fragment of a sculptured skull bone that most probably derived from a hyperossified anuran. We assign the specimens to the South American genus *Calyptocephalella*. Calyptocephalellids, or helmeted frogs, are widely known from Patagonia since the Late Cretaceous^9^. They became extinct in Argentine Patagonia during the Miocene, probably related to a decrease of humidity caused by the rise of the Andes, since the family survived to the present day in a temperate but humid refuge in the central Chilean Andes^10^.

The material described here derives from estuarine to marginal-marine deposits of the Eocene La Meseta Formation which were deposited in the James Ross Basin, a back-arc basin east of the Antarctic Peninsula, and which are widely exposed on Seymour Island^11,12^ (Fig. 1b,c). The fossil locality IAA 2/95, also known as ‘Marsupial Site’ is a few m^2^ wide lens of poorly consolidated, shelly conglomerate^8,13^. It is situated in the central portion of the *Cucullaea* I Allomember, within unit Telm 5 on the northwestern slope of the mesa (Fig. 1c,d), and informally referred to as the ‘*Natica* horizon’^8,13^. It has produced shark, ray and skate teeth, remains of marine bony fishes, as well as teeth of terrestrial mammals, worm (clitellate) cocoons, and seeds of water lilies^8,12–22^. Based on dinocyst occurrences, the age of this deposit is considered to be about 40 Ma (Bartonian, Eocene)^23,24^.

The fossil frog remains were collected during three joint Argentinian-Swedish expeditions to Seymour Island in the austral summers 2011–13. The bone fragments were concentrated from dry-sieved sediment samples as described by^8,12,20^ and sorted by using a Leica MZ6 stereomicroscope. The material is housed in the palaeozoological collections of the Swedish Museum of Natural History, Stockholm, with the inventory numbers NRM-PZ B281 and B282.

## Results

### Systematic palaeontology

Anura Fischer von Waldheim, 1813^25^

Neobatrachia Reig, 1958^26^

Australobatrachia Frost *et al*., 2006^27^

Calyptocephalellidae Reig, 1960^28^

*Calyptocephalella* Strand, 1928^29^

*Calyptocephalella* sp.

Figures 2 and 3

**Figure 2:**
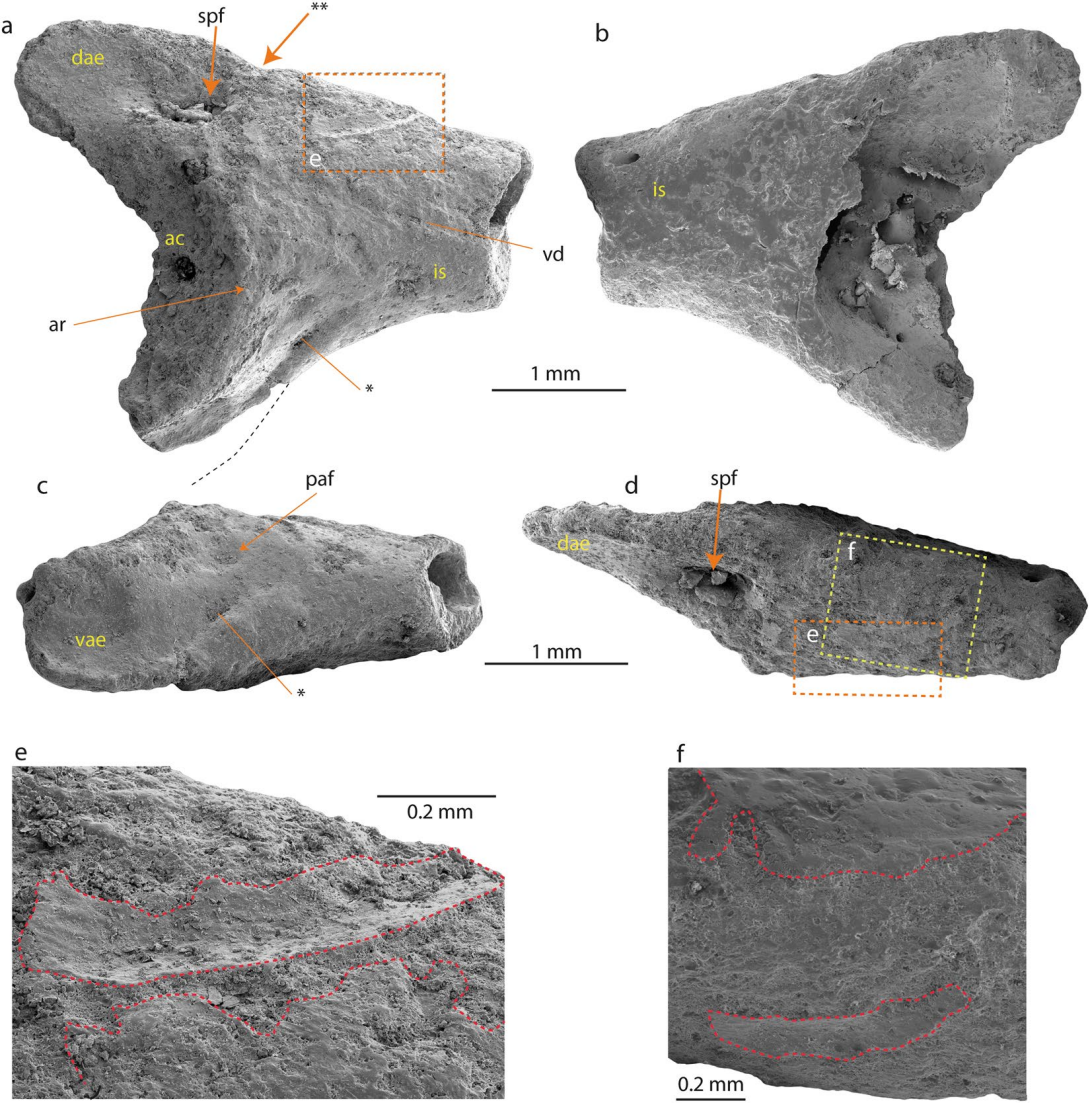
Ilium (NRM-PZ B282) of *Calyptocephalella* sp. from Seymour Island, Antarctica. Ilium in lateral (**a**), medial (**b**), ventral (**c**) and dorsal (**d**) views. Magnified region of the dorsal protuberance in lateral (**e –** dashed rectangle in orange color) and dorsal (**f –** dashed rectangle in yellow color) view. The dashed line in black on (**a**) indicates the probable outline of the posterior extension of the ventral acetabular expansion. The asterisk (*) on (**a**,**c**) indicates the shallow and broad depression of the ventral acetabular expansion. The double asterisk (**) indicates the notch caudally from the dorsal protuberance. The dashed red lines on (**e**,**f**) outline the intact bone surface. Abbreviations: ac, acetabulum; ar, acetabular rim; dae, dorsal acetabular expansion; is, iliac shaft; paf, preacetabular fossa; spf, supraacetabular fossa; vae, ventral acetabular expansion; vd, ventral depression.

**Figure 3:**
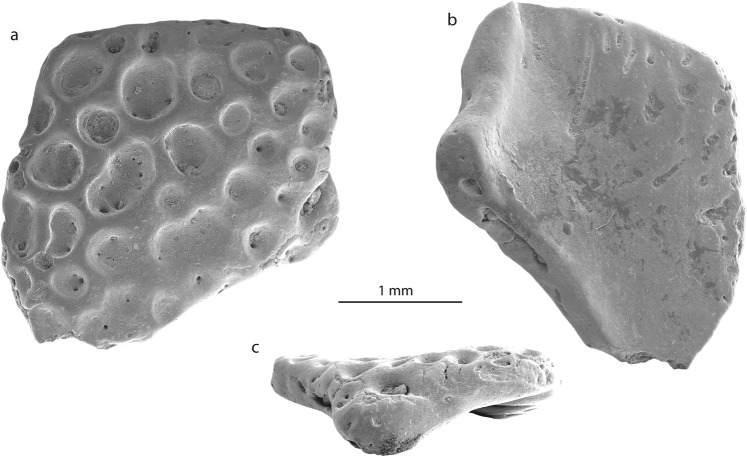
Skull bone fragment (NRM-PZ B281) of *Calyptocephalella* sp. from Seymour Island, Antarctica in dorsal (**a**), ventral (**b**) and lateral (**c**) views.

### Referred specimens

Swedish Museum of Natural History NRM-PZ B282, right ilium (Fig. 2), NRM-PZ B281, skull bone (Fig. 3).

### Locality, horizon and age

IAA 2/95, Marsupial site, Seymour Island, Antarctic Peninsula (64°13′58″S; 56°39′06″W). ‘Natica horizon’ within the Cucullaea I Allomember (Telm 5) of the La Meseta Formation, Bartonian (40 Ma), Eocene^23,24^ (Fig. 1).

### Measurements

The preserved part of the ilium measures 3.9 mm in length, the distance from the tip of the dorsal acetabular expansion to the (preserved) tip of the ventral acetabular expansion measures 3.3 mm, the highest height of the acetabular fossa equals 2.5 mm. The skull bone measures 2.7 mm at its both broadest and longest parts.

### Ilium

The fragmentary right ilium (NRM-PZ B282) lacks the caudal portion of the acetabulum and most of the iliac shaft. The dorsal acetabular expansion has a smooth lateral surface and is higher than the preserved part of the ventral acetabular expansion (Fig. 2a). A large and deep supraacetabular fossa is present at its base (Fig. 2a,d). The preserved portion of the acetabulum is concave and its shape allows concluding a (semi-)circular outline. The acetabular rim is most prominent at its anterior part (Fig. 2a,c). The barely-developed ventral acetabular expansion projects ventrally. The posterior-most portion of the ventral acetabular expansion is broken off. However, the anterior portion of the ventral acetabular expansion is higher than the preserved posterior portion. In ventral view (Fig. 2a,c), the lateral surface of the ventral acetabular expansion is convex. The ventral acetabular expansion possesses a shallow and broad depression. In the preacetabular zone, a small and shallow preacetabular fossa is present (Fig. 2c). The preserved portion of the iliac shaft is damaged and precludes a confident statement whether the dorsal protuberance is present or absent. A narrow and shallow longitudinal groove is observable in the lateral surface of the iliac shaft, which probably corresponds to the posterior extension of the ventral depression (sensu^10^) (Fig. 2a). However, intact parts of bone surface are preserved slightly ventral to the dorsal margin on both lateral and medial surfaces (Fig. 2e,f). The one on the lateral surface is a curved shallow groove and runs posteroventrally (Fig. 2e). This feature anteroventrally demarcates the slightly elevated roughened scar interpreted above as the dorsal protuberance. The area between the dorsal acetabular expansion and iliac shaft is slightly projected dorsally. This area corresponds to the position of the dorsal protuberance. In fact, no clear evidence of a dorsal protuberance can be found on the ilium, only a slightly roughened area with minimal elevation that corresponds to the dorsal protuberance and the scar for the insertion of the musculus gluteus magnus can be observed. At the caudal side of the dorsal protuberance, a distinct notch is visible (Fig. 2a) which we consider as a further evidence of our interpretation. The area corresponding to the dorsal protuberance is located anteriorly to the anterior margin of the acetabular rim. Medially, the entire surface opposing the acetabulum is lost and the area preserved more anteriorly is slightly convex medially and smooth. Anteriorly and dorsally, just adjacent to the anterior end of the dorsal protuberance a foramen is present (Fig. 2b).

**Figure 4:**
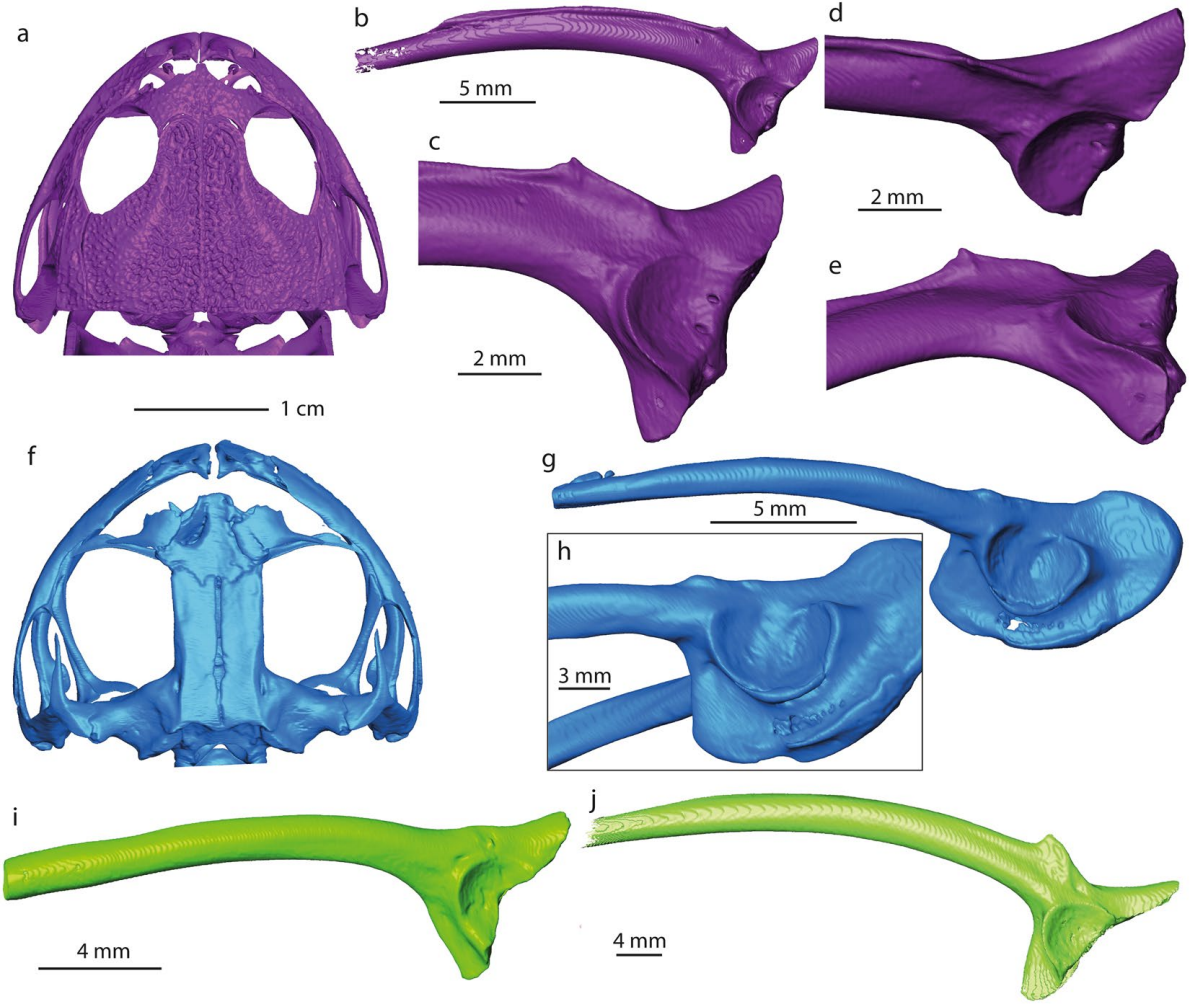
3D models of some skeletal elements of Australobatrachia. (**a,f**) skull and (**b**–**e**,**g**–**j**) ilia of *Calyptocephalella gayi* (**a–e**); *Telmatobufo venustus* (**f**–**h**); *Myobatrachus gouldii* (**i**); *Limnodynastes convexiusculus* (**j**). Collection numbers of each specimen are listed in Table S1.

The fragmentary right ilium can be referred to an anuran based on the following characters^30^ (the numbers before the characters correspond to the feature numbers of Appendix 1 in Gardner *et al*.^30^): *7*. (semi-) circular acetabulum; *9*. acetabulum with distinct margins; *10*. acetabular surface concave; *13*. at least dorsal acetabular expansion is strongly divergent; *18*. the dorsal protuberance present. Thus, the ilium derives from a small-sized frog (3.8 ± 0.4 cm snout-vent length, see methods, Table 1). The specimen is partly eroded and rather poorly preserved; however, it can be compared with all South American and Australian frog families (Figs. 4, S1 and S2, Table S1). The families Ranidae, Bufonidae and Hylidae have not been illustrated in the present work, since their morphology is well known^31^ (Table S1). The comparison has been done at family level, since the ilia display diagnostic features characteristic for identification of the family (dimensions of the dorsal and ventral acetabular expansions; location of the dorsal protuberance relative to the anterior margin of the acetabular rim etc.^32,33^). The studied ilium (NRM-PZ B282) differs in: (1) Reduced anterior portion of the dorsal acetabular expansion from nearly all South American and Australian frog families and the genus *Telmatobufo*, which have moderately or strongly developed anterior portion of the dorsal acetabular expansion. Only the genus *Calyptocephalella* (Fig. 4c,e), the families Ranidae^31^, Pipidae (Fig. S1a), Rhinodermatidae (Fig. S1j), and Leptodactylidae (Fig. S2b) have similar state/morphology of this character. (2) Dorsal protuberance located either at the level of or anteriorly from the anterior margin of the acetabular rim from nearly all families, besides Brachycephalidae (Fig. S1a), Rhinodermatidae (Fig. S1j), Telmatobiidae (Fig. S1k), Hyloididae (Fig. S1m), Leptodactylidae (Fig. S2b) and the genera *Calyptocephalella* (Fig. 4b–e) and *Telmatobufo* (Fig. 4g,h). (3) Developed dorsal acetabular expansion from the families Ranidae^31^, Hylidae^31^, Bufonidae^31^, Myobatrachidae (Fig. 4i), Pipidae (Fig. S1i), Microhylidae (Fig. S1b), Telmatobiidae (Fig. S1k), Leptodactylidae (Fig. S2b), Allophrynidae (Fig. S2c), Centrolenidae (Fig. S2d) and the genus *Telmatobufo* (Fig. 4g,h). Other families have moderately or well-developed dorsal acetabular expansion, however, due to incomplete preservation of the Antarctic frog remain any further comparison is impossible. (4) Weakly developed dorsal protuberance and lack of dorsal tubercle from nearly all families (e.g. Limnodynastidae, Fig. 4j), besides Calyptocephalellidae (Fig. 4b–e,g,h), Myobatrachidae (Fig. 4i), Craugastoridae (Fig. S1e), and Dendrabatidae (Fig. S2e).

**Table 1:** Measurements of the snout-vent length (SVL) and height of the transition from the iliac shaft and ilial body (HT) of some *Calyptocephalella* spp. and Antarctic ilia (Fig. S3), with the value of the ratio between HT and SVL (RHS = HT/SVL*100%) with the value of the standard deviation (SD).

species	locality	coll nr.	SVL (in mm)	HT (in mm)	RHS (in %)	Reference
*C. pichileufensis*	Río Pichileufú locality	BAR 85b	107	6	5.6	^48^: Figs. 2 and 4
*C. gayi*	unknown	M13105/cas:sua:10082*	54.7	2.9	5.3	^65^, see Fig. S3
range					5.3–5.6	
mean ± SD					5.45 ± 0.21	
*C*. sp.	IAA 2/95, Marsupial site	NRM-PZ B282	**38.4** ± **3.8**	**2.1**		Present work

The reconstructed value of the snout-vent length of the studied ilium is highlighted in bold.

Among the compared forms, only the South American endemic genus *Calyptocephalella* resembles all mentioned four characters. In addition to this, a shallow and broad depression on the anterior portion of the ventral acetabular expansion is a unique character observable on our ilium (NRM-PZ B282) and Recent *Calyptocephalella gayi* (Fig. 4). Further, the fossil ilium displays a ventral depression on its lateral surface anteriorly to the acetabulum (Fig. 2c), a comparable structure can be observed also in the fossil species *Calyptocephalella canqueli*^9^ but not in the Recent species *C. gayi* (Fig. 4).

### Skull element

The second bone fragment (NRM-PZ B281) is flat and slightly curved. Both sides of the bone have different structures. One surface is covered by small to large circular or reniform in outline, rather deep pits, which sink in the planar surface of the bone (Fig. 3a). The diameters of pits vary from 0.1–0.7 mm and some of them are punctured by foramina. The opposite surface of the bone is in general smooth, slightly deepened and is pierced with some foramina, some of which are preceded by a groove (Fig. 3b). One side of the fragment preserves an unbroken margin of the original bone with a distinct process that is bent and that gives the bone a curved shape (Fig. 3c). The ornamented surface of the bone projects slightly over this process. Comparable ornamentation, build of pits of different size, is found on the dorsal surfaces of different cranial and postcranial bones of amphibians and reptiles^34,35^. Among them, the following groups can be excluded from consideration: (1) Albanerpetontidae (Allocaudata); albanerpetontids are a primary Laurasian lissamphibian group with a single occurrence in Northern Africa. So far no evidence of a Gondwanan radiation of albanerpetontids exists^36^. In addition to this, all their ornamented bones (e.g. frontal, premaxillae)^37^ do not resemble the bone described here. (2) Caudata; salamanders are also considered as a Laurasian group, with a number of occurrences in Africa which need critical revision^38^. In salamanders, ornamented bones are found both among skull bones and on vertebrae (on plates located on the tip of the neural arch)^39^. Bone ornamentation here (e.g. *Tylototriton, Chelotriton, Echinotriton*^39,40^) is represented by a network of pits, ridges and pointy spines that do not resemble the bone described here. (3) Crocodylia; in crocodyliforms, comparable patterns of ornamentation with well-developed pits appear only with growth during later ontogenetic stages^41–43^. On one hand, the bone dimensions indicate a small-sized animal (corresponding to a juvenile crocodilian without such developed ornamentation). On the other hand, crocodylian osteoderms are flat without any processes, unlike the studied bone. (4) Testudines; shell plates of several turtles, such as *Trionyx, Allaeochelys etc*.^35^, are also covered by ornamentation. The ornamentation is characterized by larger and closely arranged pits, which are not always clearly delimited from each other (see Scheyer^35^: Fig. 1a). (5) Lacertilia; lizards also have skull bones and osteoderms covered with ornamentation patterns^44^. They all are characterized by a network of spines, grooves, ridges^45^ and protuberances^46^, which differs from the morphology on NRM-PZ B281.

The ornamentation pattern found in NRM-PZ B281 is comparable to that of some frog genera, i.e. *Thaumastosaurus*^47^, *Beelzebufo*^48^, *Calyptocephalella*^9^ and *Baurubatrachus*^49^, but only the last three genera are Gondwanan forms and, thus, considered for comparison herein. *Beelzebufo* is a very large form and the ornamentation pattern is present both on skull bones and on vertebrae^50^. *Calyptocephalella*^9^ and *Baurubatrachus*^49^ have very similar ornamentation patterns on the surfaces of hyperossified skull bones, comparable to our specimen. A recent phylogenetic analysis^49^ placed the Late Cretaceous *Baurubatrachus* within both Recent calyptocephalellid genera *Calyptocephalella* and *Telmatobufo*. Though Muzzopappa and Báez^10^ mention that both *Calyptocephalella* and *Telmatobufo* are characterized by a heavily ossified neurocranium, we can confirm this only for the former genus (Fig. 4a,f). Within *Calyptocephalella* the ornamentation pattern on skull bones is variable. In *C. conquella*^10^, it is built either by network of pits in small individuals, or tuberculated ornamentation in adults. In *C. satan*^9^ and *C. casamayorensis*^51^, ornamented skull bones are slightly larger than NRM-PZ B281 but they have a similar pattern built of pits. *C. pichifleufensis*^48^ is known by larger individuals which show similar ornamentation patterns but with larger pits. In comparison to these species, the Antarctic frog displays an ornamentation most similar to that of *C. satan*^9^ and *C. casamayorensis*^48^. Taking into account our comparison, we conclude that the ornamented bone fragment NRM-PZ B281 represents a skull bone (most probably a nasal) of a small-sized *Calyptocephalella* or *Baurubatrachus*. Given the presence of a small *Calyptocephalella* as indicated by the ilium in the same, only few m^2^ measuring outcrop, it is most likely that specimen NRM-PZ B281 belongs to the same genus. A comparable record of an ilium and ornamented bones referable to the genus *Calyptocephalella* has been mentioned in Báez^52^.

## Discussion

Among Recent amphibians, the frogs (Anura) have the widest distribution, covering all continents except Antarctica, where the conditions have been uninhabitable for over tens of millions of years. Contrary to all other continents, no traces of any extant amphibian group, all of which belong to the lissamphibian clade, have been documented from Antarctica. This paper presents the first record of a lissamphibian in Antarctica, with Eocene fossils referable to the order Anura, and most likely to the australobatrachian genus *Calyptocephalella*. The family Calyptocephalellidae belongs to neobatrachian frogs and is exclusively known from South America^53,54^. The five extant species, including the monospecific genus *Calyptocephalella* with hyperossified skull bones, are restricted to the Chilenean Andes^54^ while most fossil representatives are known from Argentine Patagonia^9,48,53^. Today, *Calyptocephalella* inhabits lowland areas of central Chile (upper elevation limit 500 m) east of the Andes within temperate and humid climates, between latitudes 30–43°S. It has an aquatic or semiaquatic lifestyle and populates standing or slow flowing water bodies (lakes, ponds, streams) in the Valdivian temperate *Nothofagus* forests^54–56^.

The oldest fossils referable to *Calyptocephalella* are known from the Upper Cretaceous of Argentina^9,52^. During the Paleocene–terminal early Miocene, their geographic range was restricted to Patagonia east of the Andes^48,51,53,57^. Not until the late Pleistocene did they appear west of the Andes, where they have their endemic present-day distribution^54–57^.

**Figure 5:**
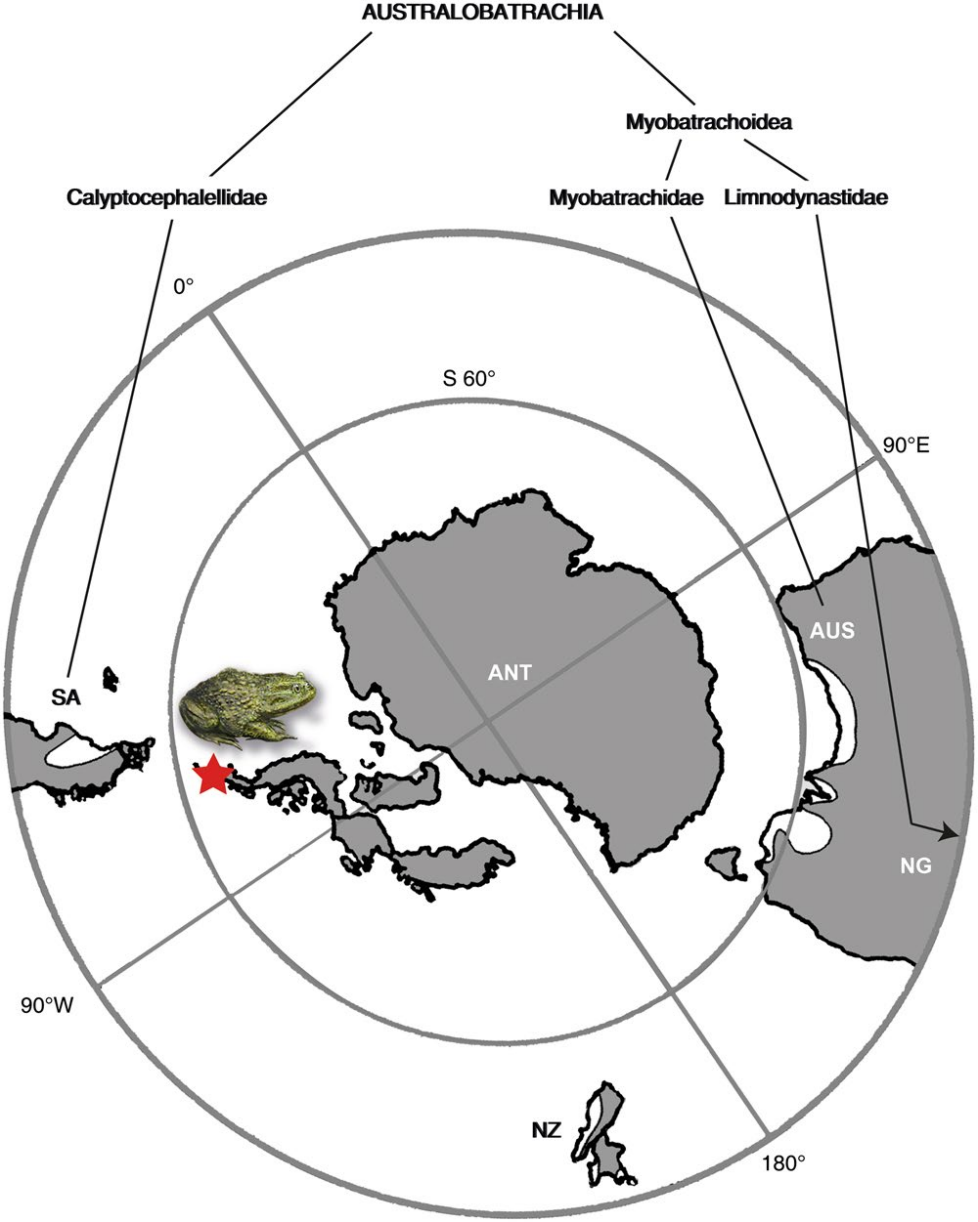
Eocene palaeogeography of the south polar region with a cladogram of australobatrachid frogs showing their occurrences on the southern continents. The grey color indicates the outlines of the continents during the Eocene, the black colored outline the present-day outlines of the continents. Map redrawn from an original generated using ArcGIS 10.17.1 (www.esri.com) software, based on the Satellite base map layer in google Maps (Map data ©2019 Google). Abbreviations: ANT, Antarctica; AUS, Australia and Tasmania; NG, New Guinea; NZ, New Zealand; SA, South America. The red star indicates the fossil locality on Seymour Island.

The clade Australobatrachia comprises Myobatrachoidea (families Myobatrachidae + Limnodynastidae sensu^27^), nowadays distributed in Australia and south of New Guinea, and the family Calyptocephalellidae (Batrachophrynidae sensu^27^). Australobatrachia are considered as a stem group of the Hyloidea. The earliest myobatrachoid from Australia is at least as old as early Eocene, based on fragmentary ilia that were referred to the basal extant *Lechriodus*^58^. The split between Calyptocephalellidae and Myobatrachidae (Calyptocephalellidae + Myobatrachoidea sensu^27^) occurred ~100 Ma (~Early-Late Cretaceous boundary)^59^. Considering the distributions of extant Australobatrachia (Fig. 5), the earliest fossil records^10^ and the divergence age (from genetic data)^59^ of both Calyptocephalellidae and Myobatrachoidea lineages, it is clear that Antarctica had played an important palaeobiogeographic role for Australobatrachia and their consequent dispersal. Because the most recent common ancestor of the clade, including Hyloidae and Myobatrachidae + Calyptocephalellidae, occurred in South America, their origin in South America and consequent dispersal from South America to Australia via Antarctica has been suggested^59^. Additionally, this suggests one more case of strong faunistic affinities of the continent with South America and Australia^4,6,16,60^. So far, Antarctica has been considered as a dispersal route, but not as a probable place of origin. The new fossil finds support the hypothesis^10^ that Antarctica may have acted as a center of diversification for australobatrachians.

**Figure 6:**
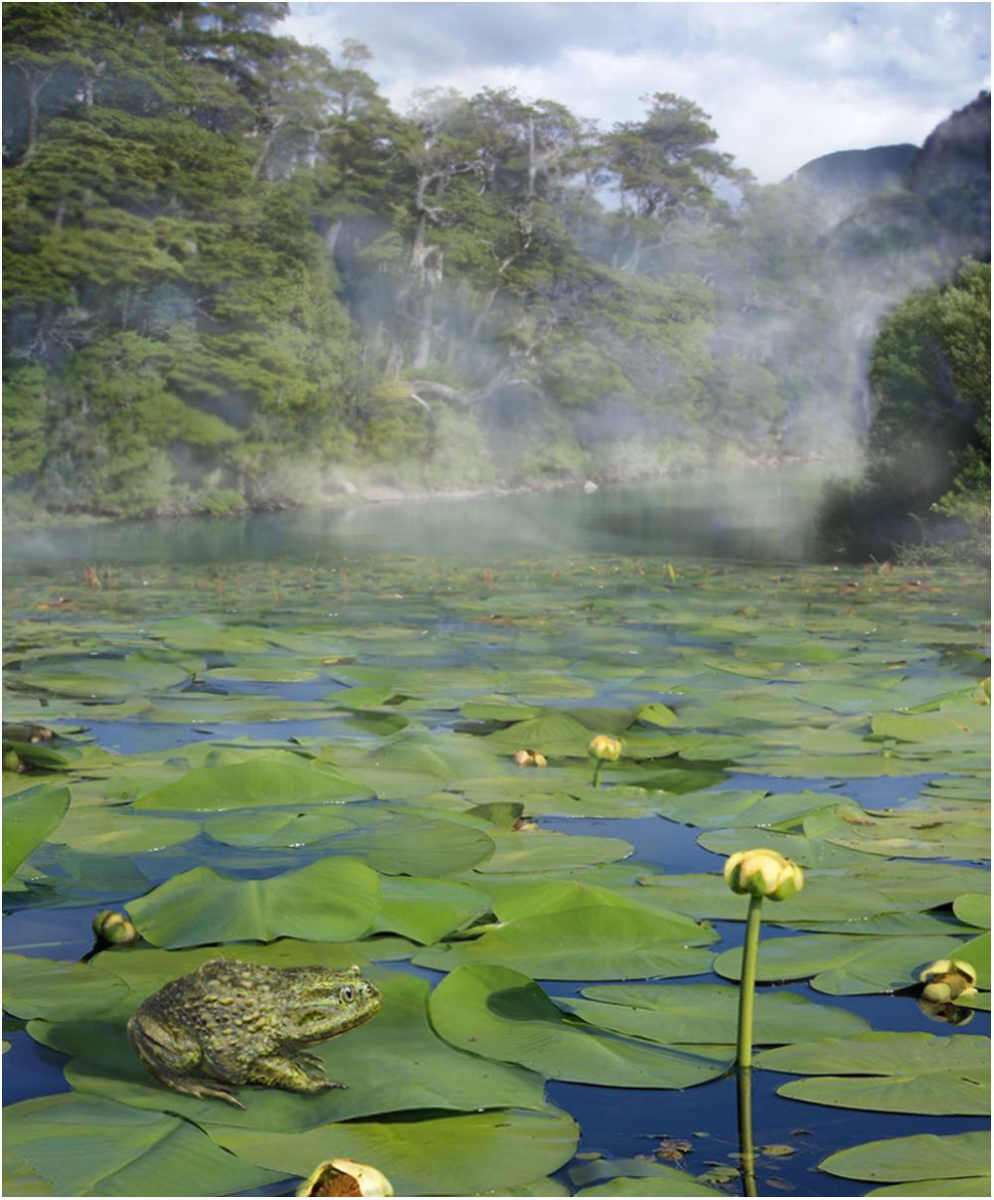
Reconstruction of an Eocene pond in the *Nothofagus* forest of the Antarctic Peninsula with *Calyptocephalella*, sitting on a leaf of *Notonuphar antarctica* which was described from the same locality^12^. Artwork by Pollyanna von Knorring, Swedish Museum of Natural History. Photo credits: Simon Pierre Barrette and José Grau de Puerto Montt, Wikimedia Commons (CC BY-SA 3.0), and Mats Wedin, Swedish Museum of Natural History.

The Seymour Island frog reported herein is the first vertebrate indicative of freshwater habitats on the Eocene Antarctic Peninsula, following invertebrate and plant evidence^12,17^ (Fig. 6). It is interesting to note that nearly all fossil localities where *Calyptocephalella* occurs (excepting those, for which fossil plant data are not available) contain evidence of the presence of *Nothofagus*, including Seymour Island^16,60,61^. The southern extant range of *Calyptocephalella* occurs sympatrically with the microbiotherian marsupial *Dromiciops gliroides* (Fig. S4), also known as “Monito del Monte” or “Colocolo Opossum”, a small mammal with an arboreal lifestyle and an endemic distribution in the dense Valdivian *Nothofagus* forests of highland Argentina and Chile^62^. The climate of this *Nothofagus* forest area with the sympatric occurrences of these two endemic animals shows humid and temperate conditions (for the numerical values, see Methods and Table 2, Fig. S4). *Dromiciops gliroides* is the only extant species of the order Microbiotheria and is considered as the only South American representative of the superorder Australidelphia which otherwise comprises Australian marsupials^63^. From the same small shell-rich lens that produced the frog remains reported herein, the fossil microbiotherian *Woodburnodon casei* has been described^64^. Hence, we hypothesize that the climatic conditions for the Antarctic Peninsula during the Bartonian (late middle Eocene) should be comparable with the climate found today in the concurrent range of the *Calyptocephalella-* and *Dromiciops*-inhabited *Nothofagus* forests of South America.

**Table 2:** Climatic parameters (MAP, MCMT, MWMT) and the elevation at the climatic stations according to the database of the word bank groups^67^.

Station number	1	2	3	4	5	6	7	8	9
City	Loanco	Nirivilo	Chillan	Antuco	Curacautin	Choshuenco	Huequi	Puerto Montt	Valdivia
Elevation (m)	70	220	127	550	541	200	30	100	100
MAP (mm)	877.82	919.5	998	1024.5	988.7	1903.53	1830	1610.8	1870
MCMT (°C)	8.74	7.9	7.69	5.27	3.75	4.51	5.04	5.57	6.9
MWMT (°C)	18.05	18.04	18.45	17.28	15.5	15	13.79	14.7	15.5

Abbreviations: MAP = mean annual precipitation, MCMT = mean coldest month temperature, MWMT = mean warmest month temperature. The location of the climatic stations can be found on Fig. S4.

The fossil finds of a frog and marsupial from Seymour Island, and their fossil and Recent distributions, represent outstanding examples of the role of global climate change on shifting biogeographic ranges. Despite global cooling and the disappearance of the habitats of these groups over large areas from Antarctica to Patagonia, they maintained their relictual occurrence in the *Nothofagus* forests of the central Chilean Andes. Thus, the Valdivian *Nothofagus* forest is a unique environment offering habitats not only for Eocene Antarctic refugees but also provides a modern analogue of the Antarctic climate just prior to the glaciation of the southern continent.

## Methods

### Documentation

The studied fossil material has been mounted on aluminum stubs, coated with gold and imaged using a Hitachi S-4300 field emission scanning-electron microscope at the Swedish Museum of Natural History (Stockholm). For the comparison with relevant families (Table S1), the CT data from the website Morphosource^65^ have been used. The visualization and segmentation of the bone material have been performed using the Amira 9.0 software in Porrentruy, Switzerland.

### Nomenclature

If not otherwise indicated, the osteological nomenclature of this study follows that of Gómez and Turazzini^66^ for the description of the fossil remains.

### Body size estimation

The values of the snout-vent length (SVL) of *Calyptocephalella* sp. from Antarctica have been reconstructed using photographs of the skeleton of *C. pichileufensis*^48^ and the 3D model of *C. gayi* (Table 1, Fig. S3). The height of the transition (HT) from the iliac shaft and ilial body (Fig. S3) have been used as reference for comparison to reconstruct the body size of NRM-PZ B282. The ratio of the HT to SVL has been used as a reference to calculate the value of the snout-vent lenth of the individual NRM-PZ B282.

### Climatic analysis

We analyzed the climatic parameters of selected stations from the area with sympatric occurrence of *Dromiciops gliroides* and *Calyptocephalella gayi* (Table 2 and Fig. S4). Since the upper elevation limit for extant *Calyptocephalella* distribution is 500 m above sea level, only stations up to this elevation have been considered for climate analysis. This analysis shows a remarkable climatic space with mean annual precipitation ≥ 900 mm, coldest month mean temperature ≥ 3.75 °C, and warmest month mean temperature ≥ 13.79 °C (Table 2). The climatic parameter variations result from both elevation and latitudinal differences, so the temperature increases and the precipitation decreases northwards, whereas an opposite trend is observable at higher altitudes.

## Supplementary information

Supplementary information.
