# Supplementary material for: First fossil frog from Antarctica: implications for Eocene high latitude climate conditions and Gondwanan cosmopolitanism of Australobatrachia
Source: Sci Rep. 2020 Apr 23;10:5051. doi: 10.1038/s41598-020-61973-5 (PMC7181706; doi:10.1038/s41598-020-61973-5)
Supplement: Supplementary file 1 — Supplementary information. [file 41598_2020_61973_MOESM1_ESM.docx]

**Title: First fossil frog from Antarctica: implications for Eocene high latitude climate conditions and Gondwanan cosmopolitanism of Australobatrachia**

**Authors**

Thomas Mörs^1,2,^*, Marcelo Reguero^3^, Davit Vasilyan^4,4^

**Affiliations**

^1^*Department of Palaeobiology, Swedish Museum of Natural History, P.O, Box 50007, SE-104 05 Stockholm, Sweden*

^2^*Bolin Centre for Climate Research, Stockholm University, Stockholm, Sweden*

^3^*Instituto Antártico Argentino, Campus Miguelete, 25 de Mayo 1151, 3° piso B1650HMK, San Martín, Buenos Aires, Argentina*

^4^*JURASSICA Museum, Fontenais 21, CH-2900 Porrentruy, Switzerland*

^5^*Department of Geosciences, University of Fribourg, Chemin du musée 6, 1700 Fribourg, Switzerland*

*Corresponding author. Email: thomas.moers@nrm.se

*
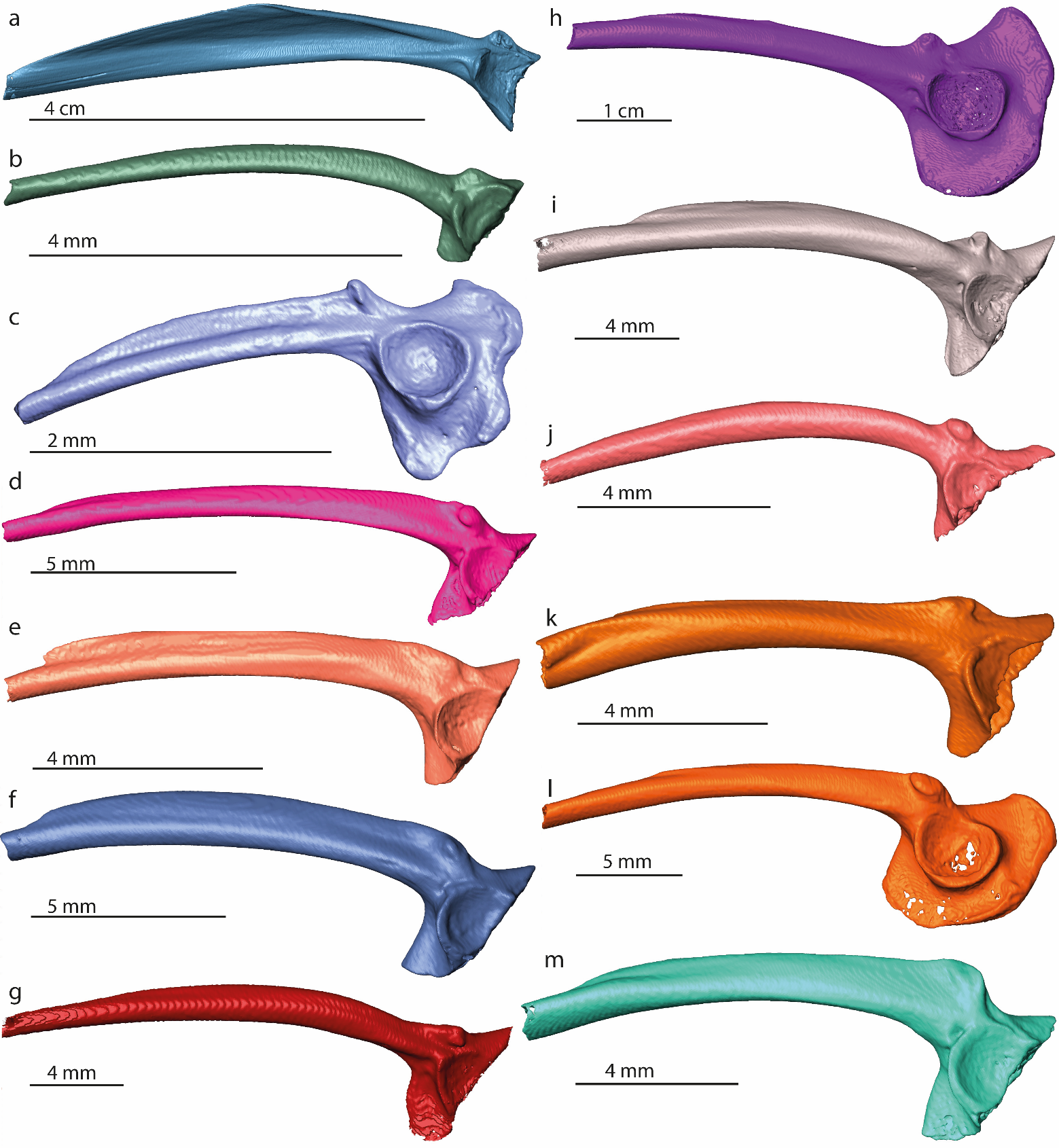
*

**Figure S1.** 3D models of ilia of *Pipa parva* (**a**), *Syncope antenori* (**b**), *Brachycephalus albolineatus* (**c**), *Eleutherodactylus glaphycompus* (**d**), *Hypodactylus araiodactylus* (**e**), *Craugastor brocci* (**f**), *Gastrotheca peruana* (**g**), *Ceratophrys aurita* (**h**), *Proceratophrys boiei* (**i**), *Rhinoderma darwinii* (**j**), *Telmatobius marmoratus* (**k**), *Cycloramphus asper* (**l**), *Hylodes asper* (**m**). Collection numbers of each specimen are listed in Table S1.


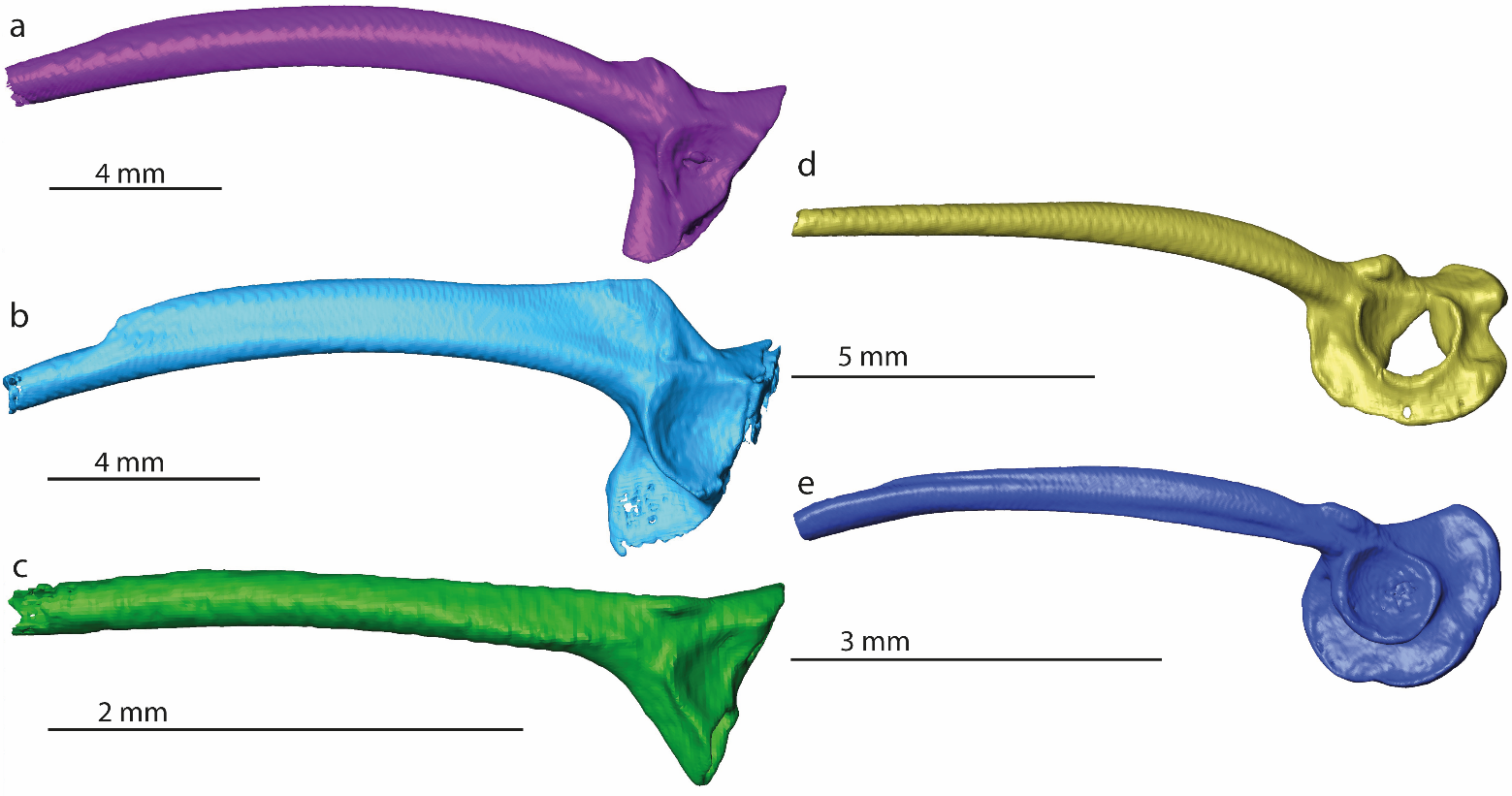


**Figure S2.** 3D models of ilia of *Alsodes nodosus* (**a**), *Leptodactylus validus* (**b**), *Allophryne ruthveni* (**c**), *Centrolene buckleyi* (**d**), *Dendrobates tinctorius* (**e**). Collection numbers of each specimen are listed in Table S1.

Table S1. List of South American and Australian frog species and families used for comparison with the Antarctic fossil. The Latin name of each species is linked to the corresponding website of Morphosource. Collection numbers and CT-scanning configurations (resolution, voltage, amperage, watts) are indicated according to the information in www.morphosource.com^1^.

| Family | Species | Figures | Collection numbers | resolution  (mm) | voltage  (kv) | amperage (µa) | watts  (W) |
| --- | --- | --- | --- | --- | --- | --- | --- |
| Pipidae | [*Pipa parva*](https://www.morphosource.org//Detail/MediaDetail/Show/media_id/18107) | Fig. S1a | M13107 / uf:herp:64234 | 0.04627829 | 120 | 200 | 24 |
| Microhylidae | [*Syncope antenori*](https://www.morphosource.org//Detail/MediaDetail/Show/media_id/25194) | Fig. S1b | M25194 / uf:herp:68008 | 0.01379663 | 80 | 150 | 12 |
| Calyptocephalellidae | [*Calyptocephalella gayi*](https://www.morphosource.org//Detail/MediaDetail/Show/media_id/13105) | Fig. 3a-e | M13105 / cas:sua:10082 | 0.03469723 | 80 | 150 | 12 |
|  | [*Telmatobufo venustus*](https://www.morphosource.org//Detail/MediaDetail/Show/media_id/22061) | Fig. 3f-h | M22061 / ku:kuh:161438 | 0.04773114 | 70 | 200 | 14 |
| Myobatrachidae | [*Myobatrachus gouldii*](https://www.morphosource.org//Detail/MediaDetail/Show/media_id/25636) | Fig. 3i | M25636 / mcz:herp:a-139543 | 0.02741526 | 80 | 200 | 16 |
| Limnodynastidae | [*Limnodynastes convexiusculus*](https://www.morphosource.org//Detail/MediaDetail/Show/media_id/25462) | Fig. 3j | M25462 / cas:herps:121263 | 0.03570469 | 70 | 200 | 14 |
| Brachycephalidae | [*Brachycephalus albolineatus*](https://www.morphosource.org//Detail/MediaDetail/Show/media_id/10212) | Fig. S1c | M10212 / MHNCI:10295 | 0.00788364 | 60 | 175 | 10.5 |
| Eleutherodactylidae | [*Eleutherodactylus glaphycompus*](https://www.morphosource.org//Detail/MediaDetail/Show/media_id/10883) | Fig. S1d | M10883 / UF:herp:56811 | 0.02484263 | 100 | 200 | N/A |
| Craugastoridae | [*Hypodactylus araiodactylus*](https://www.morphosource.org//Detail/MediaDetail/Show/media_id/13313) | Fig. S1e | M13313 / uf:40764:40764 | 0.01756294 | 100 | 250 | N/A |
|  | [*Craugastor brocci*](https://www.morphosource.org//Detail/MediaDetail/Show/media_id/25277) | Fig. S1f | M25277 / mvz:herp:264248 | 0.03366066 | 90 | 200 | 18 |
| Hemiphractidae | [*Gastrotheca peruana*](https://www.morphosource.org/Detail/MediaDetail/Show/media_id/28912) | Fig. S1g | M28912 / uf:herp:65783 | 0.04537322 | 100 | 150 | 15 |
| Ceratophryidae | [*Ceratophrys aurita*](https://www.morphosource.org/Detail/MediaDetail/Show/media_id/23249) | Fig. S1h | M10735 / cas:herp:84998 | 0.006687342 | 120 | 200 | 24 |
| Odontophrynidae | [*Proceratophrys boiei*](https://www.morphosource.org//Detail/MediaDetail/Show/media_id/13988) | Fig. S1i | M13988 / CM-H-45986 | 0.02879781 | 130 | 220 | 286 |
| Rhinodermatidae | [*Rhinoderma darwinii*](https://www.morphosource.org/Detail/MediaDetail/Show/media_id/13984) | Fig. S1j | M13975 / uf:herp:62022 | 0.026411 | 100 | 150 | 150 |
| *Telmatobiidae* | [*Telmatobius marmoratus*](https://www.morphosource.org//Detail/MediaDetail/Show/media_id/25465) | Fig. S1k | M25465 / cas:herp:152217 | 0.02721782 | 125 | 150 | 18.75 |
| *Cycloramphinae* | [*Cycloramphus asper*](https://www.morphosource.org//Detail/MediaDetail/Show/media_id/13198) | Fig. S1l | M13198 / cm:herps:68338 | 0.04225985 | 130 | 220 | 28.6 |
| Hylodidae | [*Hylodes asper*](https://www.morphosource.org//Detail/MediaDetail/Show/media_id/18125) | Fig. S1m | M18125 / cm:herps:45975 | 0.03172522 | 130 | 200 | N/A |
| Alsodidae | [*Alsodes nodosus*](https://www.morphosource.org//Detail/MediaDetail/Show/media_id/18658) | Fig. S2a | M18658 / cm:herps:63864 | 0.04678505 | 140 | 250 | 35 |
| Leptodactylidae | [*Leptodactylus validus*](https://www.morphosource.org//Detail/MediaDetail/Show/media_id/18245) | Fig. S2b | M18245 / uf:herp:103920 | 0.03359499 | 100 | 200 | 20 |
| Allophrynidae | [*Allophryne ruthveni*](https://www.morphosource.org//Detail/MediaDetail/Show/media_id/12630) | Fig. S2c | M12630 / cas:herp:257677 | 0.01428861 | 80 | 120 | N/A |
| Centroledinae | [*Centrolene buckley*](https://www.morphosource.org//Detail/MediaDetail/Show/media_id/9218)*i* | Fig. S2d | M9218 / uf:herp:30579 | 0.03331138 | 100 | 200 | N/A |
| Dendrobatidae | [*Dendrobates tinctorius*](https://www.morphosource.org//Detail/MediaDetail/Show/media_id/25692) | Fig. S2e | M25692 / ypm:vz:ypm hera 010610 | 0.01084585 | 60 | 180 | 10.8 |
| Ranidae | ^2^ |  |  |  |  |  |  |
| Bufonidae | ^2^ |  |  |  |  |  |  |
| Hylidae | ^2^ |  |  |  |  |  |  |

**
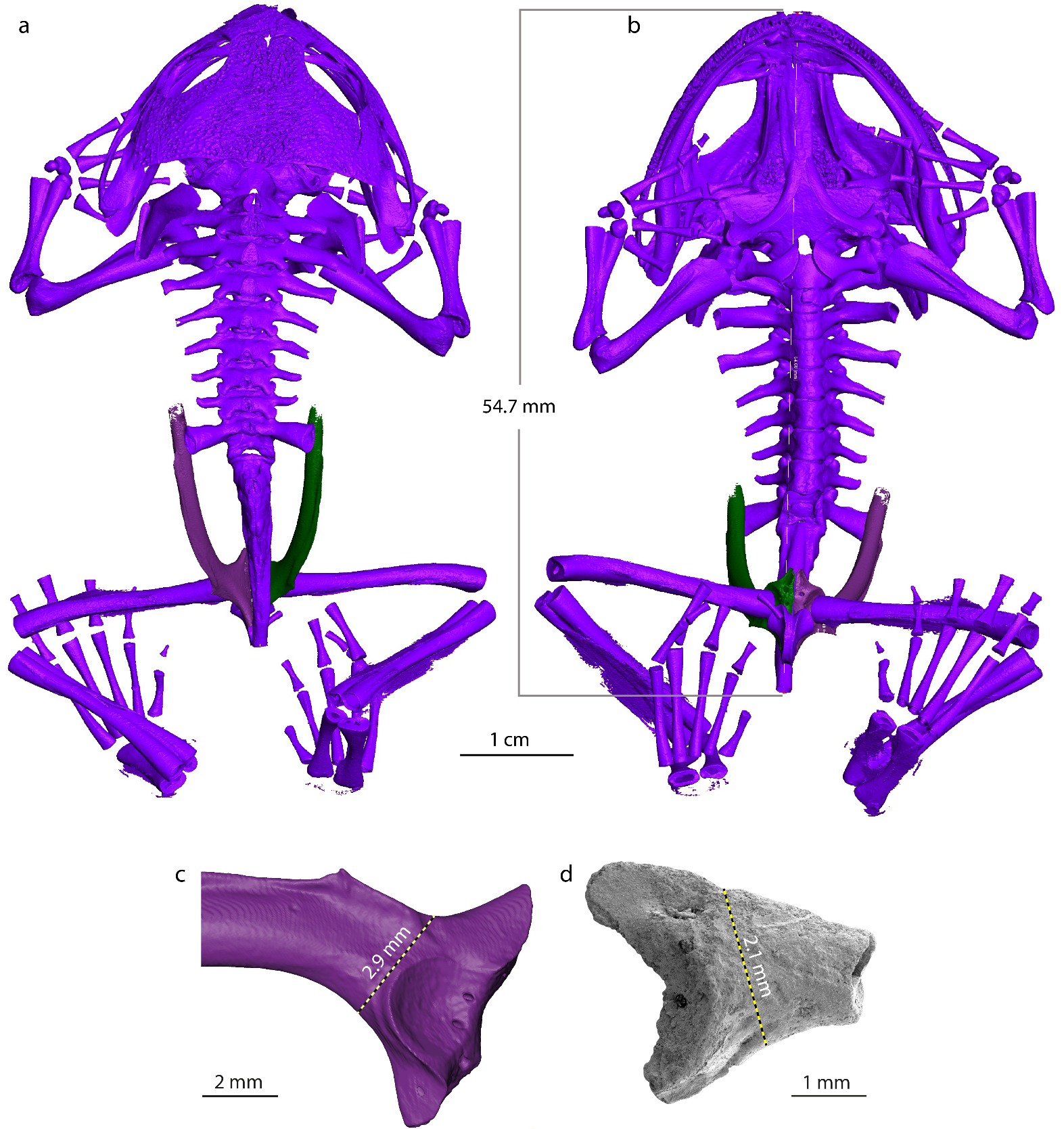
Figure S3. a-c** 3D reconstruction of *Calyptocephalella gayi* ([M13105](https://www.morphosource.org/Detail/MediaDetail/Show/media_id/13105) / cas:sua:10082 from [www.morphosourse.com](http://www.morphosourse.com)^1^). Body of *C. gayi* with indication of the measured value of the snout-vent length in dorsal (**a**) and ventral (**b**) views, as well as the left ilium of the same individual in lateral view (**c**). (**d**) Antarctic ilium (NRM-PZ B282) in lateral view. The measured distance of the height of the transition from the iliac shaft and ilial body is indicated by a black-yellow line with the corresponding numerical value.


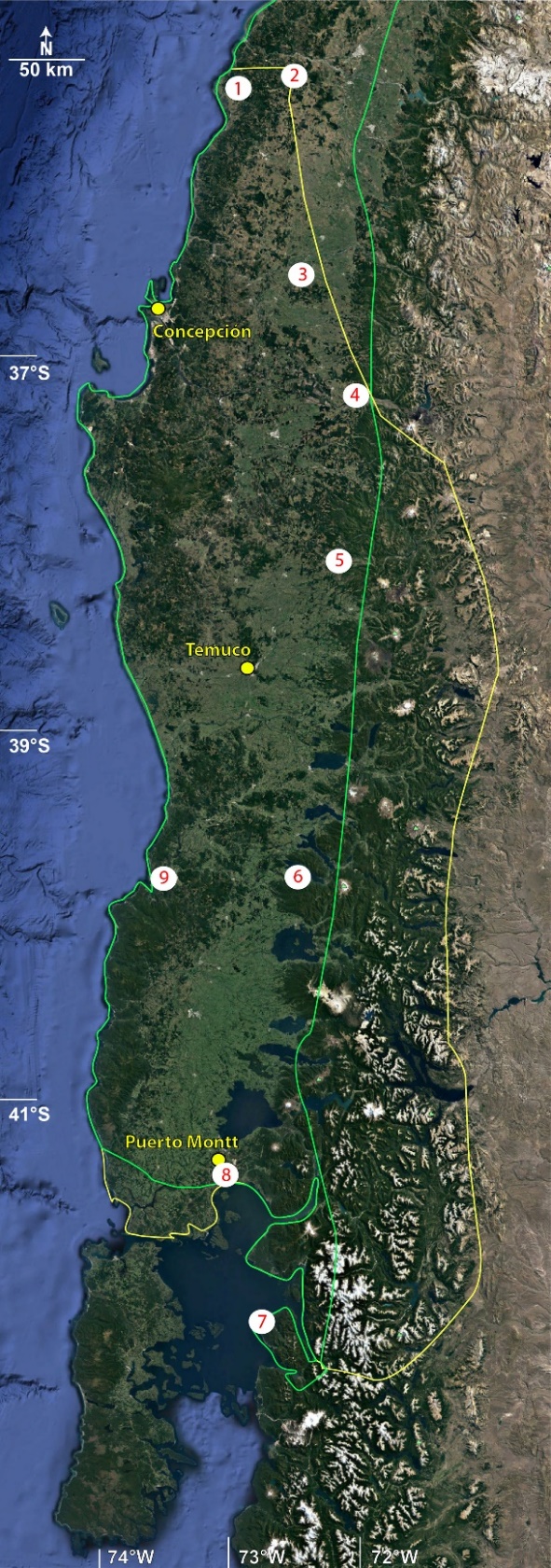


**Figure S4**. Map showing the area of the sympatric occurrences of *Calyptocephalella gayi* (green line)^3^ and *Dromiciops gliroides* (yellow line)^4^ with the locations of the climatic stations (white circles). The climatic station numbers correspond to those in Table 2. Map redrawn from an original generated using ArcGIS 10.17.1 (www.esri.com) software, based on the Satellite base map layer in google Maps (Map data ©2019 Google).

References

1. unknown. Morphosource. Available at https://www.morphosource.org/ (2020).

2. Bailon, S. *Différenciation ostéologique des Anoures (Amphibia, Anura) de France* (Centre de Recherches Archéologiques du CNRS, Antibes, 1999).

3. Veloso, A., Formas, R. J. & Gerson, H. *Calyptocephalella gayi*. The IUCN Red List of Threatened Species (2010).

4. Martin, G. M., Flores, D. & Teta, P. *Dromiciops gliroides*. IUCN Red List of Threatened Species (2015).
